# Inhibition and Production of Anger Cost More: Evidence From an ERP Study on the Production and Switch of Voluntary Facial Emotional Expression

**DOI:** 10.3389/fpsyg.2019.01276

**Published:** 2019-05-31

**Authors:** Chenyu Shangguan, Xia Wang, Xu Li, Yali Wang, Jiamei Lu, Zhizhuan Li

**Affiliations:** Department of Psychology, Shanghai Normal University, Shanghai, China

**Keywords:** voluntary facial emotional expression, production, inhibition, cost, event-related potentials

## Abstract

Humans need to flexibly produce or switch different facial emotional expressions to meet social communication need. However, little is known about the control of voluntary facial emotional expression. We investigated the production and switch of facial expressions of happiness and anger in a response-priming task of 23 Chinese female university students and recorded electroencephalographic (EEG) signals. Results revealed that a frontal-central P2 component demonstrated greater positivity in the invalidly cued condition compared with the validly cued condition. Comparing the two facial emotional expressions, data from the contingent negative variation (CNV) component revealed that happiness and anger did not differ in the motor preparation phase. While data from N2 and P3 showed that switching from anger to happiness elicited larger N2 amplitudes than switching from happiness to anger and switching from happiness to anger elicited larger P3 than switching from anger to happiness. The results revealed that in invalidly cued condition, the inhibition (N2) and reprogramming (P3) cost of anger was greater than that of happiness. The findings indicated that during the switching process, both the inhibition and the reprogramming of anger cost more processing resources than those of happiness.

## Introduction

Imagine that when you have dinner with a friend who has been bullied by his or her colleague, you get a call from your boss informing you of job promotion and a raise in salary. Will you just express your joy directly or switch the expression of happiness to anger and sadness in front of your friend? In a social context, better understanding, regulation, and expression of emotions can be essential and conducive to interpersonal interactions (e.g., Lopes et al., 2005). Previous studies focused more on the recognition and identification of facial emotional expression, whereas their production and switch were less well investigated and understood (Recio et al., 2014; Hildebrandt et al., 2015). However, it is of great importance to decrease, enhance, or switch one’s own facial expressions in certain social situations to meet the need of communicative context. In the present study, we explored the facial emotional expression from the perspective of motor control. Our aim was to investigate the neural underpinnings of the production and switch of facial emotional expressions, specifically voluntary emotional expression.

Voluntary facial emotional expressions, which are often used in social contexts, refer to those facial movements containing emotional messages that are deliberately intended or request of an individual or certain situation (Borod, 1993; Recio et al., 2014). For example, Duchenne smiles can be deliberately posed to meet certain social interaction need (Krumhuber et al., 2014). Based on social communications view, individuals can produce corresponding facial expressions due to social needs or motivations (Fridlund, 1997), and this voluntary facial expression in social contexts was considered an aspect of emotional intelligence and was crucial to individual socialization (e.g., Mayer and Salovey, 1993). In real life, individuals not only need to produce corresponding facial emotional expressions according to certain social situations but also need to switch different facial emotional expressions due to changes in communication scenarios. Meanwhile, discrete emotion theory claims that there is a small number of basic or core emotions (e.g., joy, surprise, anger), and each basic emotion has a prototypical response including an emotion-specific pattern of facial expression (Ekman, 1992; Izard, 1994). Happiness and anger are two commonly used basic emotions in human’s daily life. Therefore, in the present study, we emphasize the role of voluntary facial expressions in everyday communication and aim to explore the production and switch of voluntary facial expressions, taking expressions of happiness and anger as examples.

Comparing the facial expressions of happiness and anger, smiles need little preparation and are usually easier to control in accordance with requirements as smiles are usually encouraged and often serve communicatory functions in social interactions (e.g., Johnston et al., 2009). Smiles are usually used more frequently than other emotional expressions (Schmidt et al., 2003). In addition, previous studies of the production of facial expressions revealed that a smile involves the activation of the muscle zygomaticus, and as zygomaticus muscles are in the lower part of the face, they are easy to control voluntarily because they receive more commands from contralateral motor cortices. While the angry face involves facial muscles around eyes and these muscles receive commands from indirect corticobulbar pathway (e.g., Morecraft et al., 2004). According to the discussion above, we assumed that the voluntary expression of happiness may be more dominant than that of anger. Task-set inertia account claimed that switching from less dominant (difficult) tasks to more dominant (easy) ones requires more cognitive resources (Allport and Wylie, 2000). Because suppressing or inhibiting previous difficult task costs more cognitive resources, and recovery from suppression requires a greater cost of cognitive resources. In addition, research on attentional resources of processing different facial expressions indicated that processing of the negative expression required more attentional resources than the positive expression (e.g., Srinivasan and Gupta, 2010; Gupta and Srinivasan, 2015; Gupta et al., 2016). Therefore, the processing of angry expression may not leave enough resources to change another expression when switching from anger to happiness. While processing of happy expression requires less attentional resources, hence leaving enough resources to change another emotion. Switching from a previously intended facial expression to another involves the inhibition cost of an intended facial expression and reprogramming cost of a new facial expression. As we were interested in comparing switching from anger to happiness with switching from happiness to anger, we then assumed when switching from preparation of angry expression to execution of smile, the inhibition cost will be greater than when switching from happiness to anger as the inhibition cost of expression of anger may be larger relative to smile. Switching from anger to happiness was expected to invoke more resources than switching from happiness to anger.

Despite the importance of the production and switch of voluntary facial emotional expressions in social interactions, research investigating their neural underpinnings in experimental paradigms is still in its infancy (Korb and Sander, 2009; Hildebrandt et al., 2015). Previous studies indicated the prefrontal cortex is key to control and execute facial expression (Coan et al., 2001; Harmer et al., 2001). Event-related potentials (ERP) studies in motor program process suggested that motor preparation and switching involved contingent negative variation (CNV) component, P2, N2, and P3 components. CNV component which appeared before the response signal may reflect the preparation and expectancy of motor control (e.g., Leuthold and Jentzsch, 2002), including voluntary facial emotional expression (e.g., Recio et al., 2014; Recio and Sommer, 2018). P2 have been associated with the attentional or perceptional process and was usually larger in invalid condition than the valid condition in facial and hand motor task (Recio et al., 2014). The frontal-central N2 component could reflect the inhibition process of cognitive and motor execution as N2 amplitudes were usually larger in no-go trials in go/no-go task (e.g., Donkers and Van Boxtel, 2004). The N2 amplitudes may reflect the inhibition costs of different emotional expressions. The P3 component is a positive-going amplitude and may reflect the reprogramming of the new motor plan in voluntary emotional expressions (Recio et al., 2014; Recio and Sommer, 2018). Previous studies revealed larger N2-P3 elicited both in the anterior cingulate cortex and pre-supplementary motor area regions in a response-priming task (Leuthold and Jentzsch, 2002). A recent study investigating facial expressions of happiness and anger from the perspective of motor control found reprogramming anger induced a larger N2 and P3 (Recio et al., 2014).

The response-priming task is a powerful and excellent paradigm for studying visuomotor processing and motor control (Sterr, 2006; Schmidt et al., 2011). However, the paradigm may be less effective when mainly studying the switch of facial emotional expressions. To be specific, the paradigm often includes a high probability of valid condition (e.g., 80%) and a small probability of invalid conditions (e.g., 20%). As we stress the role of facial expressions in communication context and regard the voluntary production and switch of facial emotions as basic socioemotional competence, more emphasis will be put in investigating the switch of different facial expressions. Moreover, in some specific social communication situations or for some certain professions like the teacher, individuals often need to switch from different facial expressions according to the changing environmental requirements (Westphal et al., 2010; Yin and Lee, 2012). For example, although teachers may be in negative emotions such as sadness or anger, they still need to express excitement and happiness when they are in class. Once they find a student is not paying attention, they often need to switch from expression of happiness to an angry expression to remind the student. The ratio setting of the valid and invalid conditions of the response-priming paradigm may not be suitable to investigate the issue that individuals flexibly switch facial expressions due to social interaction changes. If the ratio of invalid conditions increased, it could increase the conflict and demand more inhibition of a prepared expression and more reprogramming of a new facial expression, which reflects the switching process. Therefore, the design in the present study increased the proportion of invalid condition (50%) to investigate the production of expressions of anger and happiness and to compare the inhibition cost of switching from expression of anger to happiness with that of switching from expression of happiness to anger.

Although previous studies have made exploratory work on the production and switch of facial expressions from the perspective of motor control with ERPs, especially research by Recio et al. (2014) and Recio and Sommer (2018), there is still a long way to go to fully understand the relevant EEG basis of voluntary facial expressions in humans. First, only a few studies explored the EEG basis of facial expressions and were mostly conducted in individualistic cultures like Western culture. Previous studies indicated the control of facial expressions could be modulated through cultural display rules or culture values (Soto et al., 2005; Butler et al., 2007; Matsumoto, 2009). Specifically, in a collectivist culture like East Asian culture, suppression or inhibition of emotion is more encouraged (Soto et al., 2005), especially regarding the expression of negative emotions like anger (Butler et al., 2007; Wei et al., 2013). Therefore, it is necessary to further explore the production and switch of voluntary facial emotional expression in East Asian culture. Second, one consensus of previous studies on emotional expression is the gender differences (Kring and Gordon, 1998; LaFrance et al., 2003). Generally, women have more expressiveness than men in facial emotional expressions (Kret and De Gelder, 2012). A meta-analysis study also concluded that women smile more than men (LaFrance et al., 2003). Based on previous studies on the gender difference in emotional expression and considering the feasibility and validity of the study, we only included women in the present study.

In the present study, we aimed to explore the potential cortical correlates of production and switch of facial expressions of happiness and anger in Chinese female participants. Especially, we were more interested in comparing the cost of switching from anger to happiness with that of switching from happiness to anger, both including the inhibition cost and reprogramming cost. Our study sought to examine the generality of the findings of Recio et al. (2014) to some degree with a different population in Eastern culture. Based on previous research (Rosenbaum, 1980; Kim et al., 2012; Recio et al., 2014), the response-priming paradigm was adapted and adopted. We expected differences in the preparation of facial expressions of anger and happiness (CNV) as they were of different emotional valence. We also predicted switching from an angry facial expression preparation to an execution of a smile elicited larger ERPs relative to switch from happy facial expression preparation to execution of angry expression. More inhibition cost and reprogramming cost of expression of anger were expected relative to expression of happiness in ERPs during the switching process.

## Materials and Methods

### Participants

Considering that men are usually less emotionally expressive than women (Kring and Gordon, 1998; Chaplin and Aldao, 2013), we chose only females in the present study. The sample size was estimated by G*power (Faul et al., 2007) with the effect size as 0.25 and power as 0.8 (see Cohen, 1988). We then aimed for a sample size of minimum 19 participants. Considering the possibility of invalid data, a total of 28 young and healthy female participants (age: 22–24 years, educational background: graduate students) enrolled in the present experiment. They had normal or corrected-to-normal vision. The study protocol was approved by the Ethical Committee of the School of Psychology at Shanghai Normal University. Informed consent was written and obtained prior to conducting the experiment and all participants received money after the experiment. We excluded one participant whose behavior performance (accuracy) was below three SDs of the overall average and four participants whose EEG signal showed excessive artifacts. The final sample consisted of 23 female participants (age: *M* = 22.73, SD = 0.69), and all participants were right-handed.

### Procedure and Stimuli

**Figure 1:**
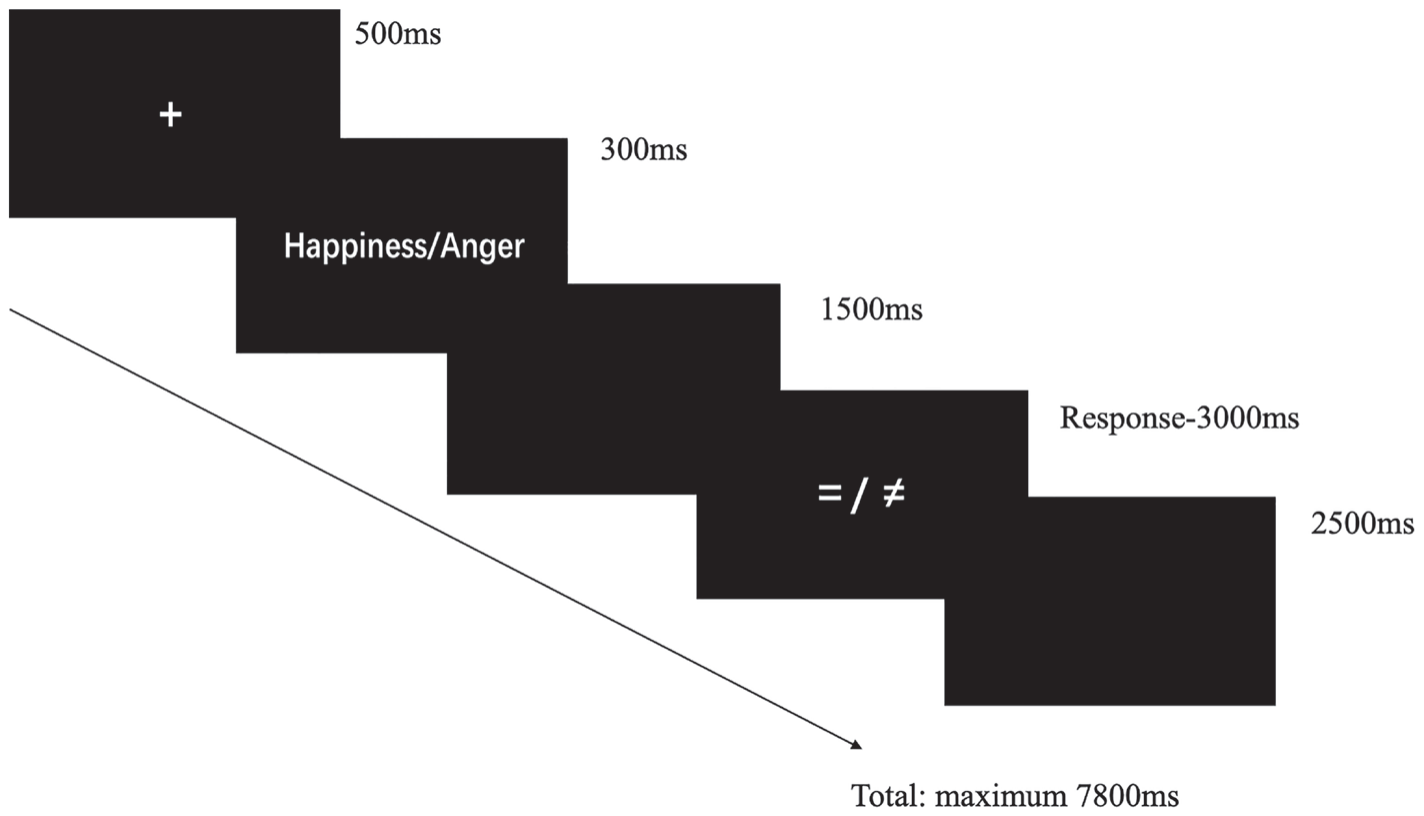
Example of one trial. The picture shows four conditions.

The experiment was administrated in a quiet chamber and the participants were at a distance of approximately 50 cm from the computer monitor. The study used the response-priming task. Figure 1 shows the structure of one experiment trial. In the task, a fixation of 500 ms was first shown and then a cue signal (happiness or anger) which lasted 300 ms was shown to remind participants to prepare the facial emotional expression mentally but to not execute before the response signal. After 1,500-ms blank screen, a response signal was shown to remind participants to execute corresponding facial emotional expressions. If the response signal was an equality symbol, the participants needed to execute the prepared expression, and immediately after the expression, participants were instructed to press keys according to the expression they just executed (“D” for happiness expression and “J” for anger expression). If the response signal was an inequality symbol, participants were instructed to execute the alternative expression, and immediately after the expression, they needed to do the same keystroke response. Therefore, there were four conditions: (1) happiness-valid condition: participants needed to prepare and produce happy expressions; (2) happiness-invalid condition: participants needed to prepare angry expression but produce happy expressions; (3) anger-valid condition: participants needed to prepare and produce angry expressions; (4) anger-invalid condition: participants needed to prepare happy expression but produce angry expressions. After the execution of facial expressions, participants were instructed to press keys according to the facial expressions they produce. If they did not press keys, the response signal will disappear after 3,000 ms. Therefore, the behavioral data including accuracy (ACC) and reaction time (RT) collected in the present study were the data collected in the response signal page. The RT included the time for the participants to execute corresponding facial expressions according to the experimental requirements and the time for them to make judgments based on their actual expressions they just executed. The ACC was calculated based on the consistency of the facial expressions that participants were required to execute under each experimental condition and the judgment based on their actual expressions. All the participants passed the practice stage to ensure that they executed the facial expression before judging. The formal experiment was organized in four blocks and there were 60 trials in each block. Happiness and anger cues were equally probable (120 trials each) while valid and invalid trials were also equally probable (120 trials each). After each block, participants were instructed to complete one item about the seriousness of executing expressions (“How conscientiously did you execute corresponding facial expressions?,” 1 = not conscientious at all, 7 = conscientious very much) and one item about the degree to press the keys as required (“Right after executing the facial expression, I press corresponding keys according to my own expression honestly,” 1 = completely no, 7 = completely yes). After finishing the two items, they had enough time to get rest. The whole process was videoed to make sure whether participants conducted the experimental tasks carefully.

### Electroencephalographic Recording and Processing

EEG signal was recorded from 64 Ag/AgCI electrodes mounted in an elastic cap (NeuroScan Inc., EI Paso, Texas, USA), sampled at 500 Hz with a bandpass filter of 0.05–100 Hz (24 dB). Impedance was maintained below 5 kΩ. Recording electrodes were referenced to the left mastoid and recalculated offline to an average mastoids reference. Vertical electrooculogram signal was measured both above and below the left eye while horizontal electrooculogram signal was measured on both sides at the external canthi. Eye-blink artifacts were rejected automatically *via* vertical ocular correction. All channels were filtered at 0.1–30 Hz offline. Incorrect response trials and artifacts exceeding ±150 μV were removed (e.g., Recio et al., 2014; Recio and Sommer, 2018). Segments were averaged in two time epochs with different conditions. For CNV: starting 100 ms before the cue signal and 800 ms after the response signal (2.7 s in total; baseline 100 ms before the onset). For P2, N2, and P3: starting 100 ms before and 800 ms after the response signal (900 ms in total; baseline 100 ms before the onset).

Based on previous voluntary facial emotional expression and related studies (Recio et al., 2014; Wu et al., 2015; Recio and Sommer, 2018) and the grand-averaged waveforms in the present study, the following ERP components, time windows, and regions of interest (ROI) were analyzed. To minimize the influence of facial movement artifacts, electrodes at frontal sites (Fp1, Fpz, and Fp2) were excluded according to Recio’s study (Recio et al., 2014). For CNV, mean amplitudes were measured in the time window of 1,600–1,900 ms (300-ms interval before the response signal) from central electrodes (FCz, Cz, C1, C2, CPz). For P2, mean amplitudes were measured in the time window of 150–210 ms from frontal-central sites (F1, Fz, F2, FC1, FCz, FC2). For N2, mean amplitudes were measured in the time window of 250–310 ms from frontal-central sites (F1, Fz, F2, FC1, FCz, FC2). For P3, mean amplitudes were measured in the time window of 330–430 ms from sites Fz, FC1, FCz, FC2, Cz, CPz, and Pz.

Repeated measures analyses of variance (rmANOVA) on the mean amplitudes of CNV, P2, N2, and P3 components were conducted with emotional expressions (two levels: happiness and anger), validity (two levels: valid and invalid), and electrodes as within-subject factors. Greenhouse-Geisser correction and Bonferroni correction were applied.

## Results

### Behavioral Results

According to results of the two-item questionnaire (seriousness of executing expressions: *M* = 6.35, SD = 0.60; making judgments right after executing expression: *M* = 6.14, SD = 0.75), and the video of the whole process, we could infer that participants completed the experiment as required. Means and standard deviations of ACC and RT for the four groups are displayed in Table 1.

**Table 1:** Means and standard deviations of ACC and RT for the four groups.

	Happiness-valid	Happiness-invalid	Anger-valid	Anger-invalid
ACC	0.99 ± 0.02	0.97 ± 0.03	0.98 ± 0.03	0.96 ± 0.06
RT/ms	1296.95 ± 259.70	1519.98 ± 270.52	1462.84 ± 256.64	1515.44 ± 248.53

*Happiness-valid condition: participants needed to prepare and produce happy expressions; happiness-invalid condition: participants needed to prepare angry expression but produce happy expressions; anger-valid condition: participants needed to prepare and produce angry expressions; anger-invalid condition: participants needed to prepare happy expression but produce angry expressions*.

Analysis of variance with repeated measures revealed a main effect of validity on ACC, *F*(1,22) = 5.02, *p* = 0.036, $\eta_{p}^{2}$ = 0.19, reflecting the ACC of valid condition significantly higher than that of invalid condition. Regarding the RT, analysis revealed a main effect of emotional expression on RT, *F*(1,22) = 7.33, *p* = 0.013, $\eta_{p}^{2}$ = 0.25. RT of angry expression condition was significantly longer than that of happy expression condition. There was a main effect of validity on RT, *F*(1,22) = 176.38, *p* < 0.001, $\eta_{p}^{2}$ = 0.89. RT of invalid condition was significantly longer than that of the valid condition. Results also revealed an interaction effect of emotional expression and validity, *F*(1,22) = 52.22, *p* < 0.001, $\eta_{p}^{2}$ = 0.70. Simple effect analysis showed that in happy expression condition, RT of invalid condition was significantly longer than that of valid condition, *F*(1,22) = 169.14, *p* < 0.001, $\eta_{p}^{2}$ = 0.89. In angry expression condition, RT of invalid condition was significantly longer than that of valid condition, *F*(1,22) = 13.88, *p* = 0.001, $\eta_{p}^{2}$ = 0.38. The validity effect was observed both in expressions of happiness and anger conditions. In order to compare the validity effect between happy and angry expression conditions, RT difference between invalid trials and valid trials was calculated respectively in both emotional expression conditions. A *t*-test was conducted and the result revealed that the RT difference was significantly longer in happy expression condition (*M* = 82.24, SD = 17.15) relative to angry expression condition (*M* = 67.72, SD = 14.12), *t*(22) = 7.23, *p* < 0.001, *d* = 3.01. Another way around analysis showed in the valid condition, there was a significant difference between happiness expression and anger expression, *F*(1,22) = 25.26, *p* < 0.001, $\eta_{p}^{2}$ = 0.53. RT of angry expression condition was significantly longer than that of happy expression condition. In the invalid condition, there was no significant difference between happiness and anger, *F*(1,22) = 0.02, *p* = 0.89.

### ERP Results

#### Contingent Negative Variation: 300-ms Interval Before the Response Signal

**Figure 2:**
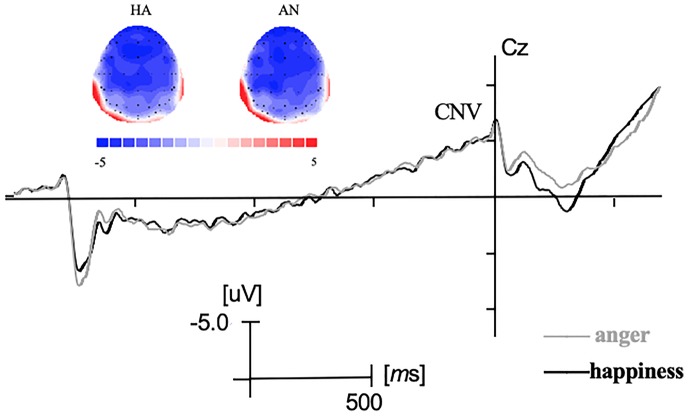
Grand-averaged ERP waveforms during the cue signal and the response signal at Cz electrode and the topographic maps during CNV time windows. HA represented the cue signal was “Happiness” and AN represented the cue signal was “Anger.”

Before the response signal, a negative-going ERP which was maximal at central electrodes resembled the CNV (see Figure 2). Analysis revealed no main effect on emotional expression and no interaction effect between emotional expression and electrodes (*F’*s < 1, *p* > 0.05).

#### P2: 150–210 ms

**Figure 3:**
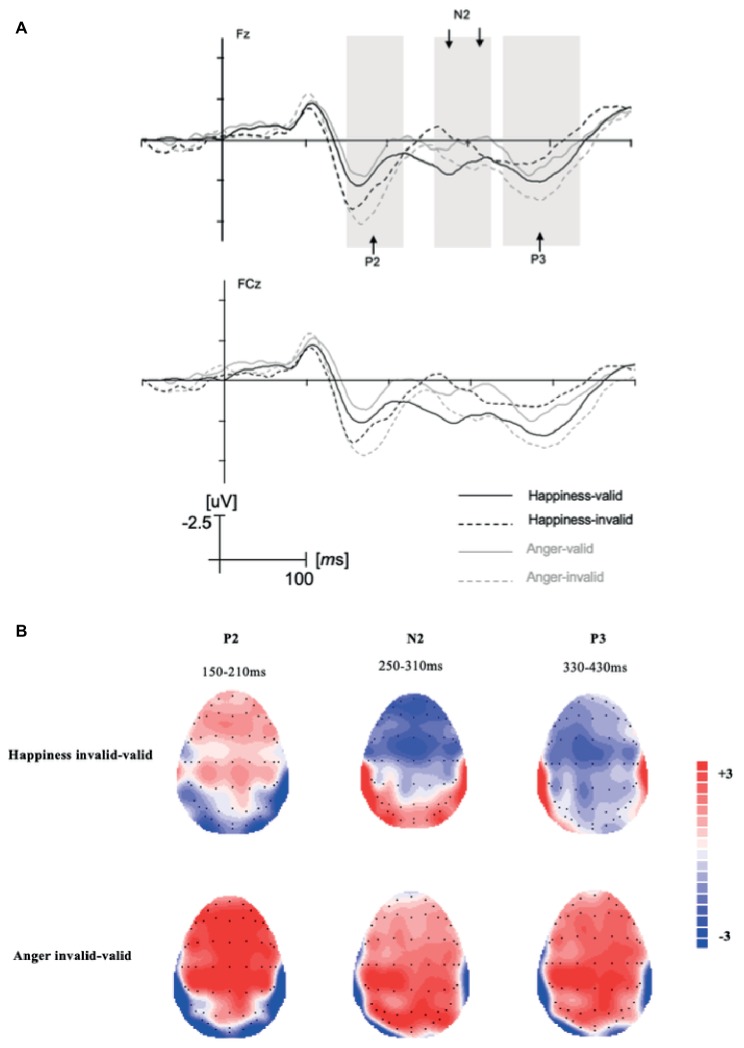
**(A)** Grand-average ERPs after the response signal at Fz and FCz with −100–500 ms time span. **(B)** The topographic maps of difference waves (invalid condition minus valid condition).

For the P2 component, there was a significant difference for validity, *F*(1,22) = 11.28, *p* = 0.003, $\eta_{p}^{2}$ = 0.34. Invalid condition (*M* = 3.48, SD = 0.64) elicited larger P2 amplitudes than valid condition (*M* = 1.86, SD = 0.61). An interaction of emotional expression and electrodes was found, *F*(5,110) = 3.04, *p* = 0.013, $\eta_{p}^{2}$ = 0.12. For happy expression, amplitudes at electrodes Fz (*M* = 2.73, SD = 0.66) were significantly larger than that at F1 (*M* = 2.49, SD = 0.65), *F*(5,18) = 2.64, *p* = 0.058, $\eta_{p}^{2}$ = 0.42; for angry expression, amplitudes at F1 electrodes (*M* = 3.02, SD = 0.61) were significantly larger than that at FC1 (*M* = 2.50, SD = 0.58), *F*(5,18) = 3.59, *p* = 0.020, $\eta_{p}^{2}$ = 0.50. As can be seen in the topographic maps in Figure 3, the P2 component in happy expression condition showed a maximum at around Fz. No main effect of emotional expression and the interaction effect was found (*F’*s < 2.75, *p’*s > 0.05).

#### N2: 250–310 ms

For N2, the interaction effect of emotional expression and validity was significant, *F*(1,22) = 6.25, *p* = 0.020, $\eta_{p}^{2}$ = 0.22. In the invalid condition, happy expression condition (*M* = 0.46, SD = 0.94) elicited larger N2 amplitudes than angry expression condition (*M* = 1.81, SD = 1.10), *F*(1,22) = 5.14, *p* = 0.034, $\eta_{p}^{2}$ = 0.19. That is, switching from anger to happiness elicited larger N2 amplitudes than switching from happiness to anger. In the valid condition, N2 amplitudes did not differ significantly, *F*(1,22) = 2.48, *p* > 0.05. Another way around simple effect analysis revealed that in happy expression condition, invalid condition (*M* = 0.46, SD = 0.94) elicited larger N2 amplitudes than valid condition (*M* = 2.31, SD = 0.78), *F*(1,22) = 8.96, *p* = 0.007, $\eta_{p}^{2}$ = 0.29. While in the angry expression condition, N2 amplitudes did not differ significantly, *F*(1,22) = 0.44, *p* > 0.05. No main effect of emotional expression, validity, and the interaction effect of the three factors was found (*F’*s < 1.71, *p’*s > 0.05).

#### P3: 330–430 ms

For P3, the interaction effect of emotional expression and validity was significant, *F*(1,22) = 11.45, *p* = 0.003, $\eta_{p}^{2}$ = 0.34. In the invalid condition, angry expression (*M* = 5.18, SD = 0.84) elicited larger P3 amplitudes than happy expression (*M* = 3.32, SD = 0.83), *F*(1,22) = 7.43, *p* = 0.012, $\eta_{p}^{2}$ = 0.25. That is, switching from happiness to anger elicited larger P3 amplitudes than switching from anger to happiness. Another way around simple effect analysis revealed that in angry expression condition, invalid trials (*M* = 5.18, SD = 0.84) elicited larger P3 amplitudes than valid trials (*M* = 3.44, SD = 0.69), *F*(1,22) = 5.54, *p* = 0.028, $\eta_{p}^{2}$ = 0.20. While in the happiness condition, invalid and valid trials did not differ significantly, *F*(1,22) = 2.80, *p* > 0.05. No main effect of emotional expression, validity, and other interaction effects was found (*F’*s < 1.59, *p’*s > 0.05).

## Discussion

In order to meet the shifting demands of communication or social context, humans need to flexibly alternate between different facial expressions of emotion. The present study aimed to investigate the neural underpinnings of motor production and switch of two facial emotional expressions (happiness and anger) and was mainly interested in comparing the inhibition and reprogramming cost of switching from anger to happiness with that of switching from happiness to anger. The present study was partly able to replicate the findings of Recio et al. (2014) with a sample of a different population in an East Asian culture. The study by Recio et al. (2014) found initial evidence supporting the greater processing resources for inhibition and reprogramming of angry expression relative to happy expression. Our results indicated that switching from an intended expression of anger to execution of expression of happiness required more inhibition cost than switching from happiness to anger. The production of a new motor plan of expression of anger also required more reprogramming cost than expression of happiness in switching process.

### Behavioral Performance

Behavioral data in the present study revealed a validity effect in the voluntary facial emotional expression field. Both in happy expression and angry expression conditions, invalid condition resulted in longer RT and lower ACC relative to valid condition, as the invalid condition involved a process of inhibiting the prepared motor (Recio et al., 2014). The result was consistent with previous studies in motor execution task (Sterr, 2006). Results in RT revealed stronger validity effect in happy expression relative to angry expression, which reflected more inhibition cost was invoked by inhibition of an intended facial movement for anger as happiness-invalid condition in the present study was pre-cued with anger. The result was consistent with previous studies exploring the control of voluntary facial expressions (Recio et al., 2014).

What we need to emphasize here is both the ACC and RT were measured by self-report measurement. The RT in the present study included both the time for the participants to execute corresponding facial expressions and the time for participants to judge which facial expression they conducted. It could not reflect the time spent in the switch phase (switch from one intended expression to the execution of another facial expression) accurately. The ACC in the present study was based on the participants’ self-reported judgments, so there may be possibilities of reporting errors. Therefore, when comparing switching from anger to happiness with switching from happiness to anger, more emphasis will be put on the ERP data analysis to compare the process in switching phase.

### ERPs

Concerning the CNV component, no significant difference was shown between the preparation progress for smiles and angry facial expression. Previous studies indicated that CNV which was maximal at the centroparietal electrodes reflected the programming and expectancy of voluntary motor action (Walter et al., 1964; Tecce, 1972; Gómez et al., 2007). The result of CNV in the present study was in line with previous studies (Recio et al., 2014), which indicated no significant difference in the preparation or the endogenous attentional effort during the expectancy of happy and angry expression in the present study. A recent study investigating the motor control over three facial expressions (smiles, disgust, and emotionally neutral jaw drops) revealed larger CNV amplitudes of neutral jaw drops than in preparing smiles or disgust expressions (Recio and Sommer, 2018). It could suggest better preparation of neutral expressions compared with emotional facial expression. One reason that there was no significant difference between expressions of happiness and anger in CNV component may be that both the expressions were emotional facial movements. It will be interesting for future studies to further investigate the neural differences between neutral and emotional facial expressions.

Validity effect appeared in the P2 component. The P2 amplitudes were larger in invalid condition for both emotional expressions. The result was consistent with previous studies (Recio et al., 2014). Previous studies indicated that the P2 component represents the perceptual-matching process and is modulated by attention (Luck and Hillyard, 1994; Freunberger et al., 2007). In the present study, the result revealed invalid condition captured more attention resource than the valid condition. It can be interpreted as the conflict or mismatch between the expectancy which the cue signal suggested and the actual facial expressions participants need to execute according to the response signal.

As for the N2 component at the frontal-central region, in happy expression condition, invalid trials elicited larger N2 amplitudes than valid trials while no such effect occurred in the angry expression condition. In addition, switching from anger to happiness elicited larger N2 amplitudes than switching from happiness to anger. Previous studies indicated that the N2 component may reflect executive cognitive control functions and executive inhibition process (Kieffaber and Hetrick, 2005). The validity effect in happy expression condition indicated more inhibition costs were invoked by inhibiting anger as happiness-invalid condition required participants to inhibit the preparation of angry expression. Meanwhile, it can be inferred that the inhibition cost of the tendency of angry expression is greater than that of happy expression. The result supported our hypothesis and is in line with previous studies in voluntary facial emotional expression (Recio et al., 2014).

Following the N2 component, larger amplitudes of the P3 component were observed for angry expression than happy expression in the invalid condition. That is, switching from happiness to anger elicited larger P3 amplitudes than switching from anger to happiness. Previous studies revealed that the more endogenous effort participants need to devote to the task, the larger the P3 amplitudes would be. P3 amplitudes were considered as a measurement of cognitive resource allocation (e.g., Isreal et al., 1980). P3 component elicited in the response-priming task could reflect the generation of a new motor plan (Recio et al., 2014). In the present study, the increase of P3 amplitudes for angry expression in invalid condition can be interpreted as the new reprogramming cost of angry expression as being greater than that of happy expression. The validity effect on angry expression condition also revealed the production of angry expression costs more processing resources than that of happy expression. It is important to point out that the validity effect was only shown in angry expression condition while no such effect occurred in happiness condition. One reason may be the relatively small P3 amplitudes in happiness-invalid condition. The smaller P3 for invalid happiness condition may result from the larger N2 in this condition, the time window of N2 component in this condition may overlap with P3 and pull the whole wave shape into less positive range. The result of P3 was consistent with the research that larger P3 was shown when processing threat-related emotional stimuli (Thomas et al., 2007), as anger is often seen as a threat-related facial expression (Green et al., 2003).

The production and switch of voluntary facial emotional expressions not only meet the changes in flexible conversation contexts but also reflect an individual’s emotional intelligence, which is of great significance to the individual’s social development. Concerning the finding that the inhibition cost of angry expression was greater relative to the happy expression during the switching phase, it can be interpreted in the perspective of evolutionary psychology to some extent, which claims that negative emotions were more critical to survival than positive emotions. Although the present study investigated the voluntary facial expressions of anger and happiness which cannot be equated with the underlying emotional states, facial emotional expressions could often convey certain emotional information. Based on embodied theories of emotion, somatic movements like voluntary facial emotional expression are closely associated with emotions (Niedenthal and Maringer, 2009). Negative emotions, like anger, fear, are considered to be products of adaptation in the process of human evolution and help individuals survive in an environment where life is threatened (Fredrickson, 2003). Many studies indicate that negative emotional stimuli capture attention faster (Pratto and John, 1991; Öhman et al., 2001) and are more difficult to escape from (Fox et al., 2001). The reason why individuals require more cognitive resources to inhibit the expression of anger, even in preparation for anger, can be interpreted in the perspective of the evolutionary significance of negative emotions to some degree. Based on the analysis on P3 amplitude, it could be inferred that the production cost of happy expression was smaller than that of angry expression. It can be interpreted as smiles are often encouraged and are more often used than other facial expressions, especially in social interactive context (e.g., Schmidt et al., 2003). In addition, the participants of the present study were all women, and previous research has shown that women smile more frequently than man (LaFrance et al., 2003), especially in social settings (Hall, 1978). In terms of social functions of facial emotional expressions, expression of happiness conveys more positive social meaning than that of anger, which can affect others’ trustworthiness to individuals (e.g., Johnston et al., 2009). Comprehensively considering why the two facial expressions (happy and angry expressions) behaved differently, it could be interpreted from the interaction of processing of positive and negative emotions with attention and perception. Previous research revealed that emotional valance interacted with attention and perception differently. Specifically, processing of positive emotional stimuli distributes attention which is directly related to global processing while processing negative emotion stimuli shrinks attention, and it is directly related to local processing (Srinivasan and Gupta, 2011). The effects of level of processing on positive and negative emotional stimuli might be linked to differences in scope of attention associated with different levels of processing. Future studies could further explore the mechanisms underlying the relationships with comparing wider range of facial expressions, including basic (e.g., excited, sad) and complex social emotional expressions (e.g., pride, shame).

Our findings may push forward the development of research in voluntary emotional expression. The results of the present study were consistent with the study of Recio et al. (2014) to some degree. The cross-cultural consistent phenomenon indicated that the production and switch of voluntary facial emotional expression may share similar neural underpinnings. One possible reason was that the present study focused on the voluntary facial expression and participants were required to execute the corresponding expression on demand, rather than spontaneously expressing or suppressing their true emotions. Future studies could further investigate the neural underpinnings of spontaneous emotional expression which is defined as those unintended movements that arise as part of an instinctual reaction to an appropriately evocative emotional simulation (Borod, 1993; Ross and Pulusu, 2012) and compare the possible difference between different cultures, as previous studies indicated different mechanisms of neural underpinnings for voluntary and spontaneous emotional expressions (Borod, 1993).

The present study has some limitations. First, the present study adopted self-reported measurement to measure facial emotional expressions of participants. This kind of measurement could not accurately reflect the time for participants to complete corresponding facial expressions and their accuracy. It is important to point out the RT in the present study included not only the time for participants to execute corresponding facial expressions but also the time for participants to judge their facial expressions. Therefore, the explanation of the RT data in the present study should be more cautious and should not be overinterpreted. In addition, although participants were required to report their actual facial expression after each trial and self-report questions were also adopted to check whether the participants did as they were required, there were still possibilities of reporting errors. So, the ACC in the present study was based on self-report measurement and also should be overinterpreted. We attributed the defects of the behavioral data in the present study to the lack of direct measurement of the facial expressions. Therefore, future studies can use more direct measurement, like Facet software. Second, only female participants were included in the present study, which limited the applicability of the results in this study. Future studies should use a larger sample which could investigate the generalization on males and the gender difference in this issue.

In summary, the present study aimed to explore the neural underpinnings of the production and switch of voluntary facial emotional expressions and was mainly interested in comparing the inhibition and reprogramming cost of switching from anger to happiness with that of switching from happiness to anger. The present study provided evidence for the validity effect in voluntary facial emotional expression, as P2 showed. In addition, comparing the expressions of happiness and anger, our data revealed the two facial emotional expressions did not differ in the preparation phase (CNV), while in the switching phase (switching from an intended expression to execution of another expression), the inhibition (N2) and reprogramming cost (P3) of anger was greater compared to expressions of happiness.

## Ethics Statement

The study protocol was approved by the Ethical Committee of the School of Psychology at Shanghai Normal University. Informed consent was obtained prior to the conduct of the experiment and all participants were get paid after the experiment.

## Author Contributions

CS contributed to conceptualization, acquisition, collection, analysis, interpretation, and drafting. XW and XL contributed to conceptualization, interpretation, and revision of the work. YW contributed to analysis and revision of the draft. JL contributed to supervision and validation. ZL contributed to revision of the draft.

### Conflict of Interest Statement

The authors declare that the research was conducted in the absence of any commercial or financial relationships that could be construed as a potential conflict of interest.
